# 3-(2-Methyl­amino-1,3-thia­zol-4-yl)-2*H*-chromen-2-one

**DOI:** 10.1107/S1600536812030140

**Published:** 2012-07-10

**Authors:** Samina Khan Yusufzai, Hasnah Osman, Aisyah Saad Abdul Rahim, Suhana Arshad, Ibrahim Abdul Razak

**Affiliations:** a School of Chemical Sciences, Universiti Sains Malaysia, 11800 USM, Penang, Malaysia; b School of Pharmaceutical Sciences, Universiti Sains Malaysia, 11800 USM, Penang, Malaysia; c School of Physics, Universiti Sains Malaysia, 11800 USM, Penang, Malaysia

## Abstract

In the title compound, C_13_H_10_N_2_O_2_S, the essentially planar 2*H*-chromene ring system [maximum deviation = 0.0297 (13) Å] and the thia­zole ring [maximum deviation = 0.0062 (11) Å] form a dihedral angle of 3.47 (5)°. In the crystal, N—H⋯N and C—H⋯O hydrogen bonds link the mol­ecules into two-dimensional networks parallel to the *bc* plane. C—H⋯π and π–π [centroid–centroid separation = 3.6796 (8) Å] inter­actions further stabilize the crystal structure.

## Related literature

For the biological activities of coumarin derivatives, see: Soine (1964 ▶); Wattenberg *et al.* (1979 ▶); Jung *et al.* (1999 ▶); Rao *et al.* (1981 ▶). For a related structure, see: Arshad *et al.* (2010 ▶, 2011 ▶); Asad *et al.* (2011 ▶); Yusufzai, Osman, Sulaiman *et al.* (2012 ▶); Yusufzai, Osman, Abdul Rahim *et al.* (2012 ▶). For the stability of the temperature controller used for data collection, see: Cosier & Glazer (1986 ▶).
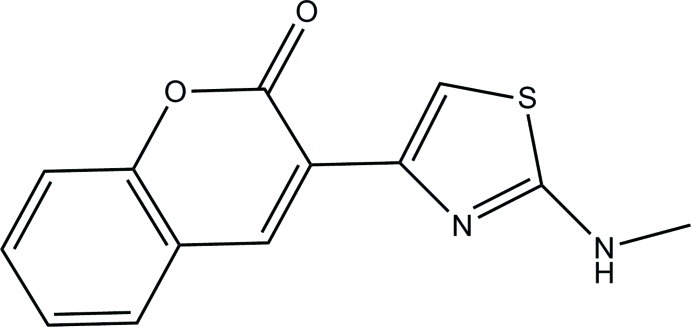


## Experimental

### 

#### Crystal data

C_13_H_10_N_2_O_2_S
*M* *_r_* = 258.29
Monoclinic, 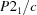

*a* = 14.5460 (3) Å
*b* = 4.9289 (1) Å
*c* = 18.3516 (3) Å
β = 120.307 (1)°
*V* = 1135.92 (4) Å^3^
*Z* = 4
Mo *K*α radiation
μ = 0.28 mm^−1^
*T* = 100 K
0.47 × 0.40 × 0.20 mm

#### Data collection

Bruker SMART APEXII CCD area-detector diffractometer
Absorption correction: multi-scan (*SADABS*; Bruker, 2009 ▶) *T*_min_ = 0.881, *T*_max_ = 0.946
13265 measured reflections
3303 independent reflections
2851 reflections with *I* > 2σ(*I*)
*R*_int_ = 0.025

#### Refinement

*R*[*F* ^2^ > 2σ(*F* ^2^)] = 0.034
*wR*(*F* ^2^) = 0.095
*S* = 1.05
3303 reflections
168 parameters
H atoms treated by a mixture of independent and constrained refinement
Δρ_max_ = 0.45 e Å^−3^
Δρ_min_ = −0.21 e Å^−3^

### 

Data collection: *APEX2* (Bruker, 2009 ▶); cell refinement: *SAINT* (Bruker, 2009 ▶); data reduction: *SAINT*; program(s) used to solve structure: *SHELXTL* (Sheldrick, 2008 ▶); program(s) used to refine structure: *SHELXTL*; molecular graphics: *SHELXTL*; software used to prepare material for publication: *SHELXTL* and *PLATON* (Spek, 2009 ▶).

## Supplementary Material

Crystal structure: contains datablock(s) global, I. DOI: 10.1107/S1600536812030140/rz2775sup1.cif

Structure factors: contains datablock(s) I. DOI: 10.1107/S1600536812030140/rz2775Isup2.hkl

Supplementary material file. DOI: 10.1107/S1600536812030140/rz2775Isup3.cml

Additional supplementary materials:  crystallographic information; 3D view; checkCIF report

## Figures and Tables

**Table 1:** Hydrogen-bond geometry (Å, °) *Cg*1 is the centroid of the S1/N1/C10–C12 ring.

*D*—H⋯*A*	*D*—H	H⋯*A*	*D*⋯*A*	*D*—H⋯*A*
N2—H1N2⋯N1^i^	0.87 (2)	2.24 (2)	3.0331 (15)	152 (2)
C4—H4*A*⋯O2^ii^	0.95	2.44	3.3247 (16)	154
C13—H13*C*⋯*Cg*1^iii^	0.98	2.70	3.5026 (16)	139

Symmetry codes: (i) 
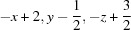
; (ii) 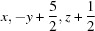; (iii) 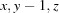.

**Fig. 1.**
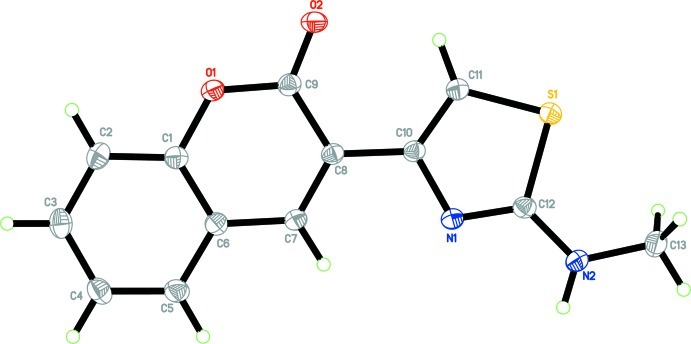
The molecular structure of the title compound, showing 50% probability displacement ellipsoids.

**Fig. 2.**
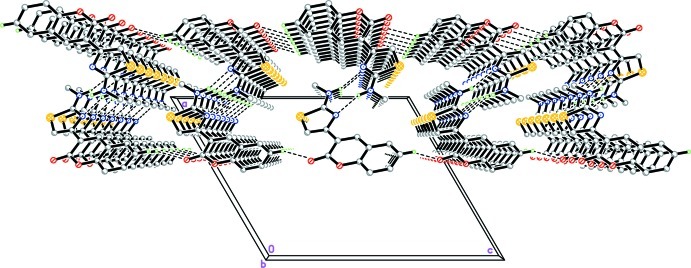
The crystal packing of the title compound, viewed down the b axis. The H atoms not involved in the intermolecular interactions (dashed lines) have been omitted for clarity.

## supplementary crystallographic information

### Comment

Compounds containing the coumarin moiety exhibit useful and diverse biological activity and, in recent years, there has been a growing interest in their synthesis (Soine, 1964; Wattenberg *et al.*, 1979). Some of these coumarin derivatives have been found to be useful in photochemotherapy, antitumour, anti-HIV therapy (Jung *et al.*, 1999), as antibacterial (Rao *et al.*, 1981) and as anticoagulant (Jung *et al.*, 1999). In continuation of our previous work (Yusufzai, Osman, Sulaiman *et al.*, 2012; Yusufzai, Osman, Abdul Rahim *et al.*, 2012) we have synthesized 3-(2-methylamino-1,3-thiazol-4-yl)-3,4-dihydro-2*H*-chromen-2-one, a new compound which corresponds to the molecular formula C_13_H_10_N_2_O_2_S. Its melting point was found to be 192–194 °C. The structure of the newly synthesized compound was confirmed by its spectral data. Synthesis of other derivatives of coumarinthiourea and their biological activities are under progress.

The molecular structure of the title compound is shown in Fig. 1. The 2*H*-chromene ring (O1/C1–C9) and the thiazole ring (S1/N1/C10–C12) are essentially planar with maximum deviations of 0.0297 (13) Å at atom C7 and 0.0062 (11) Å at atom N1, respectively. The dihedral angle between the 2*H*-chromene and thiazole rings is 3.47 (5)°. Bond lengths and angles are within normal ranges and are comparable to those found in related structures (Arshad *et al.*, 2010; Arshad *et al.*, 2011; Asad *et al.*, 2011).

In the crystal packing (Fig. 2), the intermolecular N2—H1N2···N1 and C4—H4A···O2 (Table 1) hydrogen bonds link the molecules into a two dimensional network parallel to *bc* plane. C13—H13C···*Cg*1 (Table 1) interactions and π–π interactions [*Cg*1···*Cg*2^iii^ = 3.6796 (8) Å; symmetry code: (iii) x, -1+y, z] further stabilize the crystal structure (*Cg*1 and *Cg*2 are the centroids of the S1/N1/C10–C12 and O1/C1/C6–C9 rings, respectively).

### Experimental

To a solution of 3-(2-bromoacetyl)-2*H*-chromen-2-one (0.001 mol) in absolute ethanol (20 mL), *N*-methylthiourea (0.001 mol) was added with stirring. The reaction mixture was refluxed for 3–4 hours. The precipitate formed on slow evaporation of solvent was collected by filtration, washed with cold ethanol and dried under vacuum. Recrystallization by ethanol gave the title compound as orange crystals.

### Refinement

The N-bound H atom was located in a difference Fourier map and refined freely [N–H = 0.87 (2) Å]. The remaining H atoms were positioned geometrically [C–H = 0.95 or 0.98 Å] and refined using a riding model with *U*_iso_(H) = 1.2 or 1.5 *U*_eq_(C). A rotating group model was applied to the methyl group.

### Crystal data

**Table:**

C_13_H_10_N_2_O_2_S	*F*(000) = 536
*M**_r_* = 258.29	*D*_x_ = 1.510 Mg m^−^^3^
Monoclinic, *P*2_1_/*c*	Mo *K*α radiation, λ = 0.71073 Å
Hall symbol: -P 2ybc	Cell parameters from 7338 reflections
*a* = 14.5460 (3) Å	θ = 2.6–32.5°
*b* = 4.9289 (1) Å	µ = 0.28 mm^−^^1^
*c* = 18.3516 (3) Å	*T* = 100 K
β = 120.307 (1)°	Block, orange
*V* = 1135.92 (4) Å^3^	0.47 × 0.40 × 0.20 mm
*Z* = 4	

### Data collection

**Table:**

Bruker SMART APEXII CCD area-detector diffractometer	3303 independent reflections
Radiation source: fine-focus sealed tube	2851 reflections with *I* > 2σ(*I*)
Graphite monochromator	*R*_int_ = 0.025
φ and ω scans	θ_max_ = 30.0°, θ_min_ = 1.6°
Absorption correction: multi-scan (*SADABS*; Bruker, 2009)	*h* = −20→16
*T*_min_ = 0.881, *T*_max_ = 0.946	*k* = −6→6
13265 measured reflections	*l* = −25→25

### Refinement

**Table:**

Refinement on *F*^2^	Primary atom site location: structure-invariant direct methods
Least-squares matrix: full	Secondary atom site location: difference Fourier map
*R*[*F*^2^ > 2σ(*F*^2^)] = 0.034	Hydrogen site location: inferred from neighbouring sites
*wR*(*F*^2^) = 0.095	H atoms treated by a mixture of independent and constrained refinement
*S* = 1.05	*w* = 1/[σ^2^(*F*_o_^2^) + (0.0472*P*)^2^ + 0.5573*P*] where *P* = (*F*_o_^2^ + 2*F*_c_^2^)/3
3303 reflections	(Δ/σ)_max_ = 0.001
168 parameters	Δρ_max_ = 0.45 e Å^−^^3^
0 restraints	Δρ_min_ = −0.21 e Å^−^^3^

### Special details

**Table:**

Experimental. The crystal was placed in the cold stream of an Oxford Cryosystems Cobra open-flow nitrogen cryostat (Cosier & Glazer, 1986) operating at 100.0 (1) K.
Geometry. All e.s.d.'s (except the e.s.d. in the dihedral angle between two l.s. planes) are estimated using the full covariance matrix. The cell e.s.d.'s are taken into account individually in the estimation of e.s.d.'s in distances, angles and torsion angles; correlations between e.s.d.'s in cell parameters are only used when they are defined by crystal symmetry. An approximate (isotropic) treatment of cell e.s.d.'s is used for estimating e.s.d.'s involving l.s. planes.
Refinement. Refinement of *F*^2^ against ALL reflections. The weighted *R*-factor *wR* and goodness of fit *S* are based on *F*^2^, conventional *R*-factors *R* are based on *F*, with *F* set to zero for negative *F*^2^. The threshold expression of *F*^2^ > σ(*F*^2^) is used only for calculating *R*-factors(gt) *etc*. and is not relevant to the choice of reflections for refinement. *R*-factors based on *F*^2^ are statistically about twice as large as those based on *F*, and *R*- factors based on ALL data will be even larger.

### Fractional atomic coordinates and isotropic or equivalent isotropic displacement parameters (Å^2^)

**Table:**

	*x*	*y*	*z*	*U*_iso_*/*U*_eq_	
S1	0.86394 (2)	0.35198 (6)	0.510226 (18)	0.01927 (9)	
O1	0.59607 (7)	1.21689 (19)	0.55233 (5)	0.02025 (19)	
O2	0.62018 (7)	1.0170 (2)	0.45629 (6)	0.0226 (2)	
N1	0.87142 (8)	0.5208 (2)	0.64688 (6)	0.0167 (2)	
N2	0.99500 (9)	0.1750 (2)	0.66837 (7)	0.0209 (2)	
C1	0.61748 (9)	1.2526 (3)	0.63378 (7)	0.0174 (2)	
C2	0.56079 (10)	1.4526 (3)	0.64782 (8)	0.0215 (2)	
H2A	0.5090	1.5594	0.6026	0.026*	
C3	0.58198 (10)	1.4917 (3)	0.72954 (9)	0.0223 (3)	
H3A	0.5447	1.6288	0.7405	0.027*	
C4	0.65730 (10)	1.3329 (3)	0.79635 (8)	0.0220 (3)	
H4A	0.6707	1.3618	0.8520	0.026*	
C5	0.71206 (10)	1.1340 (3)	0.78079 (8)	0.0207 (2)	
H5A	0.7626	1.0245	0.8259	0.025*	
C6	0.69360 (9)	1.0923 (3)	0.69871 (7)	0.0170 (2)	
C7	0.74892 (9)	0.8943 (2)	0.67830 (7)	0.0172 (2)	
H7A	0.8000	0.7812	0.7219	0.021*	
C8	0.73070 (9)	0.8631 (2)	0.59846 (7)	0.0157 (2)	
C9	0.64806 (9)	1.0288 (3)	0.53044 (7)	0.0175 (2)	
C10	0.79048 (9)	0.6641 (2)	0.57920 (7)	0.0159 (2)	
C11	0.77572 (10)	0.6015 (3)	0.50172 (7)	0.0188 (2)	
H11A	0.7242	0.6834	0.4503	0.023*	
C12	0.91583 (9)	0.3476 (2)	0.61957 (7)	0.0167 (2)	
C13	1.04154 (10)	−0.0065 (3)	0.63373 (8)	0.0228 (3)	
H13A	1.0934	−0.1250	0.6785	0.034*	
H13B	1.0773	0.0999	0.6101	0.034*	
H13C	0.9854	−0.1171	0.5891	0.034*	
H1N2	1.0201 (16)	0.177 (4)	0.7226 (13)	0.040 (5)*	

### Atomic displacement parameters (Å^2^)

**Table:**

	*U*^11^	*U*^22^	*U*^33^	*U*^12^	*U*^13^	*U*^23^
S1	0.01899 (15)	0.02255 (16)	0.01429 (14)	0.00258 (11)	0.00693 (11)	−0.00217 (10)
O1	0.0194 (4)	0.0245 (4)	0.0164 (4)	0.0064 (3)	0.0087 (3)	0.0043 (3)
O2	0.0213 (4)	0.0301 (5)	0.0154 (4)	0.0051 (4)	0.0085 (3)	0.0045 (4)
N1	0.0147 (4)	0.0188 (5)	0.0143 (4)	0.0008 (4)	0.0056 (4)	0.0000 (4)
N2	0.0185 (5)	0.0242 (5)	0.0156 (5)	0.0060 (4)	0.0054 (4)	−0.0004 (4)
C1	0.0162 (5)	0.0193 (5)	0.0171 (5)	−0.0007 (4)	0.0087 (4)	0.0005 (4)
C2	0.0183 (5)	0.0208 (6)	0.0254 (6)	0.0035 (5)	0.0110 (5)	0.0022 (5)
C3	0.0199 (6)	0.0215 (6)	0.0296 (6)	−0.0006 (5)	0.0155 (5)	−0.0035 (5)
C4	0.0196 (6)	0.0268 (6)	0.0212 (6)	−0.0020 (5)	0.0114 (5)	−0.0054 (5)
C5	0.0168 (5)	0.0266 (6)	0.0172 (5)	0.0008 (5)	0.0075 (4)	−0.0012 (5)
C6	0.0136 (5)	0.0193 (5)	0.0171 (5)	−0.0009 (4)	0.0070 (4)	−0.0010 (4)
C7	0.0148 (5)	0.0197 (5)	0.0149 (5)	0.0019 (4)	0.0058 (4)	0.0007 (4)
C8	0.0137 (5)	0.0168 (5)	0.0154 (5)	−0.0001 (4)	0.0064 (4)	0.0011 (4)
C9	0.0151 (5)	0.0196 (5)	0.0177 (5)	0.0008 (4)	0.0082 (4)	0.0017 (4)
C10	0.0138 (5)	0.0173 (5)	0.0149 (5)	−0.0002 (4)	0.0061 (4)	0.0001 (4)
C11	0.0181 (5)	0.0208 (6)	0.0154 (5)	0.0029 (4)	0.0068 (4)	0.0000 (4)
C12	0.0145 (5)	0.0183 (5)	0.0151 (5)	−0.0017 (4)	0.0058 (4)	−0.0014 (4)
C13	0.0191 (5)	0.0237 (6)	0.0235 (6)	0.0037 (5)	0.0092 (5)	−0.0028 (5)

### Geometric parameters (Å, º)

**Table:**

S1—C11	1.7265 (13)	C3—H3A	0.9500
S1—C12	1.7517 (12)	C4—C5	1.3807 (17)
O1—C1	1.3757 (14)	C4—H4A	0.9500
O1—C9	1.3781 (15)	C5—C6	1.4054 (16)
O2—C9	1.2094 (15)	C5—H5A	0.9500
N1—C12	1.3129 (15)	C6—C7	1.4296 (16)
N1—C10	1.3971 (15)	C7—C8	1.3598 (16)
N2—C12	1.3457 (16)	C7—H7A	0.9500
N2—C13	1.4482 (16)	C8—C10	1.4674 (16)
N2—H1N2	0.87 (2)	C8—C9	1.4683 (16)
C1—C2	1.3903 (17)	C10—C11	1.3621 (16)
C1—C6	1.3923 (16)	C11—H11A	0.9500
C2—C3	1.3838 (18)	C13—H13A	0.9800
C2—H2A	0.9500	C13—H13B	0.9800
C3—C4	1.4001 (19)	C13—H13C	0.9800
			
C11—S1—C12	89.04 (6)	C8—C7—C6	122.02 (11)
C1—O1—C9	123.10 (9)	C8—C7—H7A	119.0
C12—N1—C10	110.22 (10)	C6—C7—H7A	119.0
C12—N2—C13	122.07 (11)	C7—C8—C10	121.21 (10)
C12—N2—H1N2	118.6 (13)	C7—C8—C9	118.93 (11)
C13—N2—H1N2	119.3 (13)	C10—C8—C9	119.84 (10)
O1—C1—C2	117.51 (11)	O2—C9—O1	116.01 (11)
O1—C1—C6	120.24 (11)	O2—C9—C8	126.56 (11)
C2—C1—C6	122.25 (11)	O1—C9—C8	117.43 (10)
C3—C2—C1	118.10 (12)	C11—C10—N1	115.57 (11)
C3—C2—H2A	121.0	C11—C10—C8	127.05 (11)
C1—C2—H2A	121.0	N1—C10—C8	117.38 (10)
C2—C3—C4	121.28 (12)	C10—C11—S1	110.40 (9)
C2—C3—H3A	119.4	C10—C11—H11A	124.8
C4—C3—H3A	119.4	S1—C11—H11A	124.8
C5—C4—C3	119.60 (12)	N1—C12—N2	125.28 (11)
C5—C4—H4A	120.2	N1—C12—S1	114.75 (9)
C3—C4—H4A	120.2	N2—C12—S1	119.97 (9)
C4—C5—C6	120.49 (12)	N2—C13—H13A	109.5
C4—C5—H5A	119.8	N2—C13—H13B	109.5
C6—C5—H5A	119.8	H13A—C13—H13B	109.5
C1—C6—C5	118.27 (11)	N2—C13—H13C	109.5
C1—C6—C7	118.21 (11)	H13A—C13—H13C	109.5
C5—C6—C7	123.52 (11)	H13B—C13—H13C	109.5
			
C9—O1—C1—C2	178.87 (11)	C7—C8—C9—O2	−177.09 (12)
C9—O1—C1—C6	−0.99 (17)	C10—C8—C9—O2	1.73 (19)
O1—C1—C2—C3	−179.64 (11)	C7—C8—C9—O1	2.65 (16)
C6—C1—C2—C3	0.21 (19)	C10—C8—C9—O1	−178.53 (10)
C1—C2—C3—C4	−0.76 (19)	C12—N1—C10—C11	−1.08 (15)
C2—C3—C4—C5	0.3 (2)	C12—N1—C10—C8	178.73 (10)
C3—C4—C5—C6	0.75 (19)	C7—C8—C10—C11	175.76 (12)
O1—C1—C6—C5	−179.36 (11)	C9—C8—C10—C11	−3.03 (19)
C2—C1—C6—C5	0.79 (18)	C7—C8—C10—N1	−4.03 (17)
O1—C1—C6—C7	0.75 (17)	C9—C8—C10—N1	177.18 (10)
C2—C1—C6—C7	−179.10 (11)	N1—C10—C11—S1	0.53 (14)
C4—C5—C6—C1	−1.27 (18)	C8—C10—C11—S1	−179.26 (10)
C4—C5—C6—C7	178.62 (12)	C12—S1—C11—C10	0.09 (10)
C1—C6—C7—C8	1.30 (18)	C10—N1—C12—N2	−178.77 (12)
C5—C6—C7—C8	−178.59 (12)	C10—N1—C12—S1	1.14 (13)
C6—C7—C8—C10	178.23 (11)	C13—N2—C12—N1	−178.67 (12)
C6—C7—C8—C9	−2.98 (18)	C13—N2—C12—S1	1.43 (17)
C1—O1—C9—O2	179.07 (11)	C11—S1—C12—N1	−0.74 (10)
C1—O1—C9—C8	−0.70 (16)	C11—S1—C12—N2	179.17 (11)

### Hydrogen-bond geometry (Å, º)

Cg1 is the centroid of the S1/N1/C10–C12 ring

**Table:**

*D*—H···*A*	*D*—H	H···*A*	*D*···*A*	*D*—H···*A*
N2—H1*N*2···N1^i^	0.87 (2)	2.24 (2)	3.0331 (15)	152 (2)
C4—H4*A*···O2^ii^	0.95	2.44	3.3247 (16)	154
C13—H13*C*···*Cg*1^iii^	0.98	2.70	3.5026 (16)	139

Symmetry codes: (i) −*x*+2, *y*−1/2, −*z*+3/2; (ii) *x*, −*y*+5/2, *z*+1/2; (iii) *x*, *y*−1, *z*.
